# Supplementary material for: Interaction between Myricetin Aggregates and Lipase under Simplified Intestinal Conditions
Source: Foods. 2020 Jun 11;9(6):777. doi: 10.3390/foods9060777 (PMC7353558; doi:10.3390/foods9060777)
Supplement: Supplementary file 1 [file foods-09-00777-s001.pdf]

Supplementary information

1. Raw turbidity results over two hours of incubation

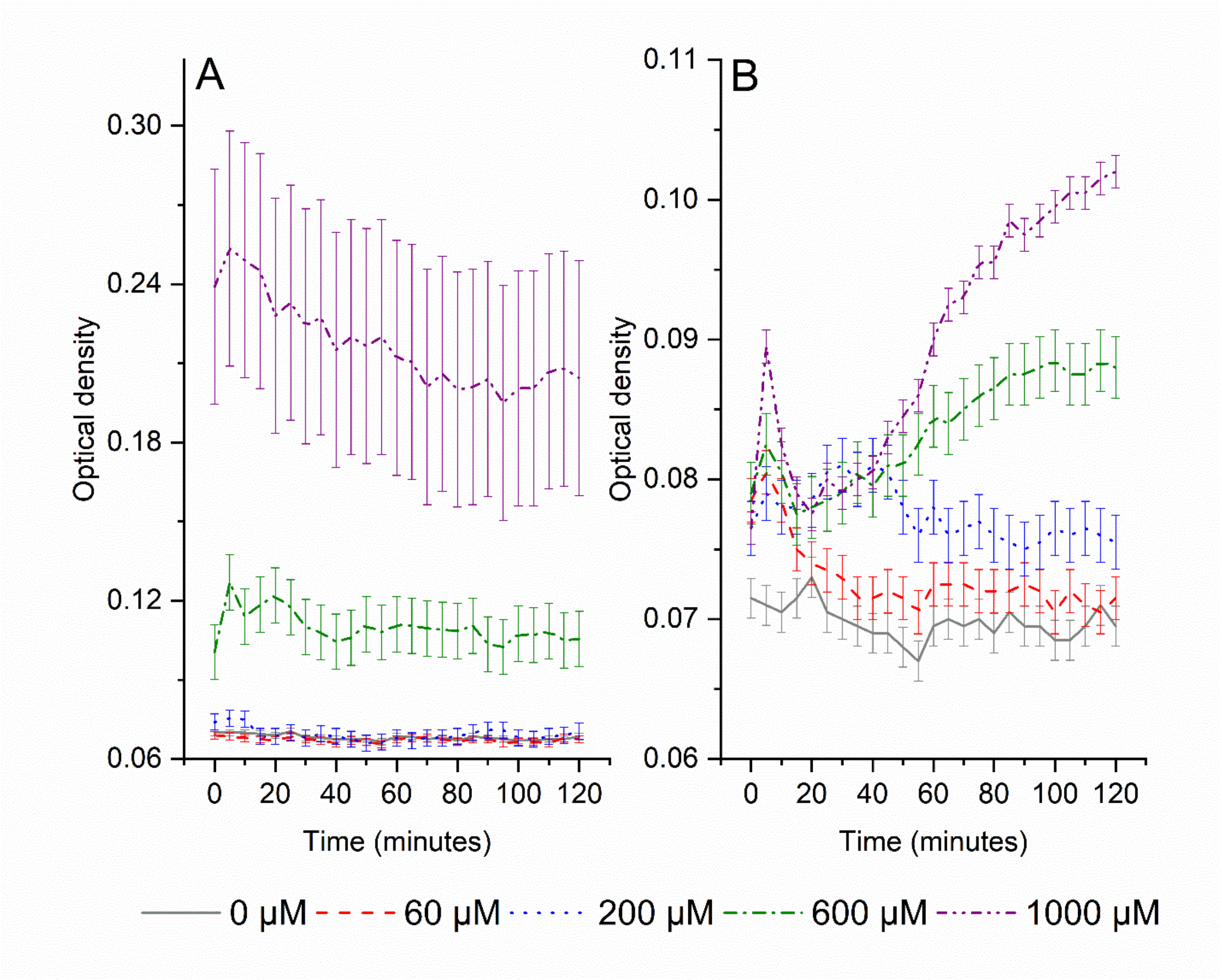

Figure S1. Aggregation kinetics of myricetin under different conditions for 5 different concentrations. (**A**) Myricetin—water samples, (**B**) Myricetin—intestinal solution samples. The error bars represent the pooled standard deviation from duplicates.

2. Molecular mass calculation from AF4

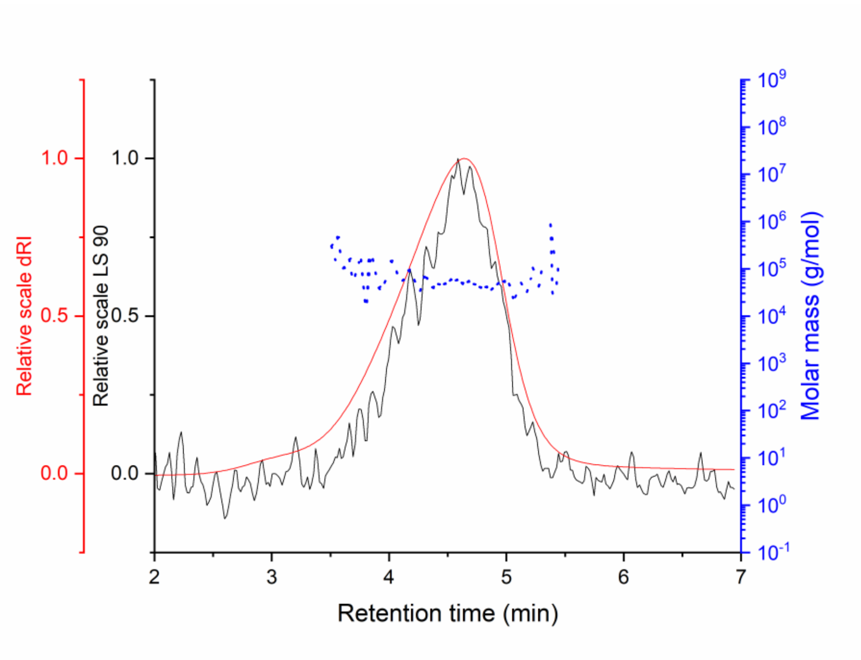

Figure S2. AF4–MALS–dRI fractograms. Average molar mass 50 kDa. See section *AF4 data processing* for more information.

3. Molecular dynamic simulations

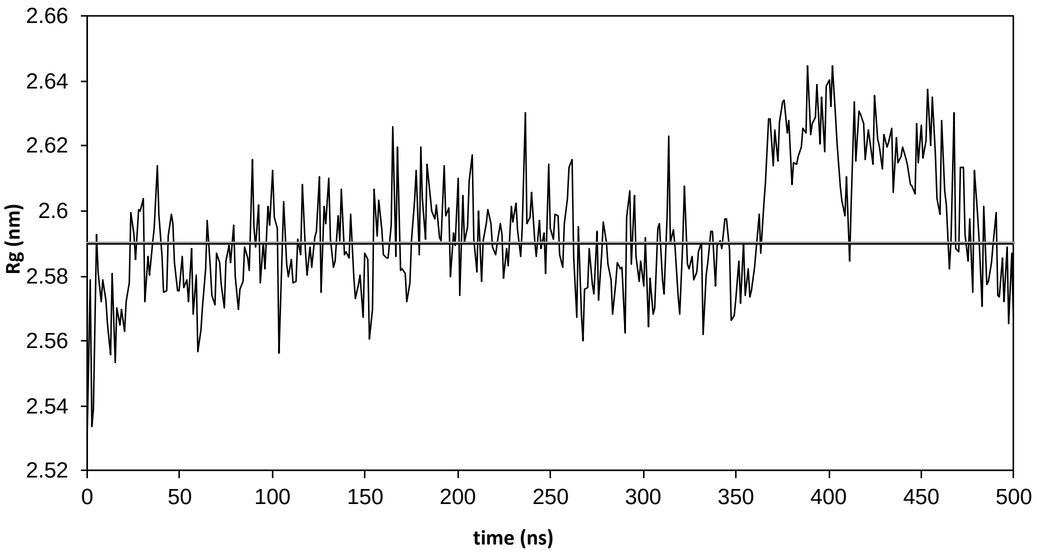

Figure S3. Radius of gyration obtained in MD analysis for the porcine lipase.
